# An In Vitro and In Vivo Assessment of the Anti‐Breast Cancer Activity of *Erythrina excelsa* Baker (Fabaceae) Bark Extracts

**DOI:** 10.1002/cnr2.70642

**Published:** 2026-09-01

**Authors:** Jonas Walantini, Marius Trésor Kemegne Sipping, Merline Nguedia Ymele, Roland Nhouma Rebe, Joël Abel Gbaweng Yaya, Derek Tantoh Ndinteh, Dieudonné Njamen, Stéphane Zingue

**Affiliations:** ^1^ Department of Biological Sciences, Faculty of Science University of Maroua Maroua Cameroon; ^2^ Department of Animal Biology and Physiology, Faculty of Science University of Yaoundé 1 Yaoundé Cameroon; ^3^ Department of Chemistry, Faculty of Science University of Ngaoundere Ngaoundere Cameroon; ^4^ Centre for Natural Product Research, Department of Chemical Sciences University of Johannesburg Johannesburg South Africa; ^5^ Department of Pharmacotoxicology and Pharmacokinetics, Faculty of Medicine and Biomedical Sciences University of Yaoundé 1 Yaoundé Cameroon

**Keywords:** anti‐inflammatory, antioxidant, apoptosis, breast cancer, cell cycle, DMBA, *Erythrina excelsa*

## Abstract

**Background:**

Breast cancer remains a topic of interest due to its high mortality rate among Cameroonian women. The barks of *Erythrina excelsa* are traditionally used for medicinal purposes in the management of various conditions including cancer.

**Aims:**

This study evaluated the phytochemical composition, antioxidant properties, and anticancer effects of *Erythrina excelsa* bark extract in vitro and in vivo.

**Methods and Results:**

Phytochemical profiling and antioxidant assays (DPPH, FRAP) were conducted to determine secondary metabolite content and free radical scavenging capacity. Cytotoxicity, apoptosis, and cell cycle arrest were assessed in MCF‐7 and MDA‐MB‐231 breast cancer cell lines. In vivo chemopreventive efficacy was evaluated using a DMBA‐induced mammary carcinogenesis in 42 female rats aged 55 to 65 days (~100 g), except the normal group (*n* = 7). The normal (NOR) and negative (DMBA) groups were treated daily with the vehicle (2% ethanol in distilled water) while the positive (Tamox) and test groups received tamoxifen (3.3 mg/kg) and *Erythrina excelsa* extract (75, 150, 300 mg/kg BW), respectively for 20 weeks. Tumor parameters, serum biomarkers (CA 15–3, cytokines), oxidative stress markers, hematological and biochemical indices, and organ toxicity were assessed. As results, both aqueous (AE) and ethanolic (EE) extracts of *E. excelsa* inhibited the growth of MCF‐7 and MDA‐MB‐231 breast cancer cells in a dose‐dependent manner after 24 h, induced apoptosis, and promoted G2/M cell cycle arrest. In vivo, *E. excelsa* aqueous extract (AE) significantly reduced tumor incidence, volume, and weight in a DMBA‐induced mammary carcinogenesis rat model, while lowering serum CA15‐3 and pro‐inflammatory cytokines (IFN‐γ, TNF‐α, IL‐6, IL‐12). Treatment also enhanced antioxidant defenses (SOD, catalase, GSH), reduced lipid peroxidation (MDA), improved hematological and biochemical parameters, and mitigated DMBA‐induced hepatic and renal toxicity, without evidence of systemic side effects.

**Conclusion:**

*Erythrina excelsa* barks exhibited an anticancer potential mediated by its antioxidant, anti‐inflammatory, pro‐apoptotic, and cell cycle–modulating effects, and support its further investigation as a promising natural source for cancer therapy.

**Abbreviations**
ALT: alanine transaminase
AST: aspartate aminotransferase
BSA: bovine serum albumin
CA 15–3: cancer antigen 15–3
DMBA: 7,12‐dimethylbenz(a) anthracene
DNA: deoxyribonucleic acid
ELISA: enzyme‐linked immunosorbent assay
EMT: epithelial to mesenchymal transition
FBS: fetal bovine serum
FRAP: ferric reducing antioxidant power
HRP: horseradish peroxidase
IFN‐γ: interferon‐gamma
IL: interleukins
JNK: N‐terminal kinase c‐Jun
MDA: malondialdehyde
NHC: national herbarium of cameroon
PAH: polycyclic aromatic hydrocarbons
PBS: phosphate‐buffered saline
PI: propidium iodide
RBCs: red blood cells
ROS: reactive oxygen species
RT: room temperature
SERM: selective estrogen receptor modulator
TFC: total flavonoid content
TNF‐α: tumor necrosis factor alpha
VEGF: vascular endothelial growth factor
WBC: white blood cells

## 1 Introduction

Cancer persists as a major contemporary health concern. This condition affects millions worldwide and ranks as the second cause of mortality after cardiovascular diseases. In 2020 alone, approximately 19.3 million new cases were diagnosed and around 10 million lives were lost [1]. In the absence of major advances, it is predicted that this number may rise to about 17 million annual deaths by 2030 [2]. Cancer occurs when cells become unresponsive to the body's regulatory mechanisms, evade programmed cell death, and proliferate uncontrollably [2]. Over time, the cells invade the nearby tissues, subsequently spreading to distant organs and forming metastases, responsible for 90% of deaths [3]. A critical component of this process is angiogenesis, defined as the capacity of tumors to generate self‐sufficient blood supply to sustain subsequent proliferation [3]. The etiology of cancer is multifactorial, involving a combination of genetic susceptibility, environmental influences and lifestyle factors. These include obesity, smoking, radiation exposure, chronic stress, physical inactivity, and inherited mutations in genes such as BRCA1 and BRCA2. Hormonal (such as early menarche and late menopause) and environmental (e.g., exposure to polycyclic aromatic hydrocarbons such as DMBA) factors also play a role in the development of disease [4].

Among the various forms of cancer, breast cancer is the leading cause of cancer‐related mortality in women. On a global scale, it accounted for 2.3 million new cases and nearly 685 000 deaths in 2020, representing 30% of all cancer cases diagnosed globally in women [2]. In the geographical region of sub‐Saharan Africa, breast cancer has been observed to cause a particularly high mortality rate. For instance, in Cameroon, the annual incidence of the disease is reported to be more than 4000 new cases, with a mortality of over 2000 women [5]. Treatment regimens generally consist of a combination of surgical interventions, chemotherapy, immunotherapy, hormone therapy, or targeted pharmacotherapy. Some chemotherapeutic agents may disrupt DNA and cell division processes to selectively eliminate cancer cells [6]. Tamoxifen, classified as a selective estrogen receptor modulator (SERM), competitively blocks the binding of estradiol to estrogen receptors, thereby impeding the receptor's interaction with the estrogen response element on DNA [7]. While these approaches have demonstrated efficacy, it is imperative to acknowledge their inherent limitations. Drugs such as tamoxifen often result in damage to healthy organs (liver and kidneys), increasing the risk of secondary cancers, and are associated with drug resistance, tumor recurrence, and their high financial costs [8]. In the context of resource‐limited settings, the intersection of these barriers with late diagnosis engenders particularly challenging environment for survival [9]. These challenges have prompted researchers and communities to explore alternative solutions. Across Africa and Asia, a significant proportion of the population, estimated up to 80%, relies on medicinal plants for the management of a wide range of health conditions, including cancer [10]. Herbal remedies are known to be more accessible, affordable, and less toxic than conventional treatments, and they remain an essential part of healthcare for many. One plant of growing interest is *Erythrina excelsa*, Baker for its richness in bioactive compounds like long‐chain acid ester and an isoflavonoid prenyl [11]. Moreover, in Uganda and Cameroon, *E. excelsa* is widely grown as an ornamental and roadside tree. Local communities in the South‐West region of Cameroon have historically employed *E. excelsa* to manage menopausal symptoms and treat gynecological growths such as ovarian cysts and uterine fibroids [12, 13]. Guided by these traditional uses and preliminary phytochemical findings, the present study seeks to examine the potential of *E. excelsa* bark extracts in the context of breast cancer.

## 2 Materials and Methods

### 2.1 Chemicals

The following chemicals and reagents have been used in this study.

**Table:** 

No.	Substances	Roles	Provider
1	7,12‐dimethylbenz(a)anthracene (DMBA, purity ≥ 98%)	Breast carcinogen	Sigma‐Aldrich (Stanford, Germany)
2	Tamoxifen Citrate (Norvatis Access)	Standard (positive control)	Salutas Pharma GmbH (Barbelen, Germany).
3	3‐(4,5‐dimethylthiazol‐2‐yl)‐2,5‐diphenyl tetrazolium bromide (MTT)	Cell growth	Roche Diagnostics (Penzberg, Germany)
5	Diazepam (Valium 10 mg/2 mL)	Anaesthetic	Roche (Fontenay‐sous‐Bois, France)
6	Ketamine (ketamine hypochloride 50 mg/mL)	Anaesthetic	Rotex Medica (Tritau, Germany)
7	Bovine fetal serum (FBS), GlutaMax, penicillin, streptomycin	Cell culture	Gibco/Invitrogen (Karlsruhe, Germany)
8	Annexin V‐FITC Apoptosis Detection Kit	Apoptosis Detection	BD Pharmingen (Heidelberg, Germany)
9	The Sandwich Cytokine Assay Kit for rat	Determination of cytokine levels	Milipore (R&D Systems Inc., Minneapolis, USA)
10	The Cancer Antigen 15–3 (CA 15–3) ELISA Kit	Breast Cancer Biomarker	Monobind Inc. (California, USA).
11	Activity kits ALT, AST, urea, creatinine, triglycerides and cholesterol	Colometric assay	Bio‐diagnostics Co. (Giza, Egypt).

### 2.2 Plant Material

#### 2.2.1 Harvesting and Extraction Procedure

The stem barks of *Erythrina excelsa* was collected in Ngaoundere 1, located in the Adamawa region of Cameroon. Botanical authentication was performed at the National Herbarium of Cameroon (HNC‐IRA, Yaoundé) by Mr. NANA, through comparison with a voucher specimen deposited at reference number No. 61487 HNC.

The collected bark of the trunk of *E. excelsa* was cut into small pieces, air‐dried at room temperature, and pulverized to a fine powder. Then, 2000 g of the powdered material was macerated in 6 L of distilled water for 24 h. The suspension was further filtered through Whatman N° 4 filter paper. The aqueous filtrate was concentrated by oven‐drying at 50°C for 24 h, and the resultant extract was preserved at 4°C until use. This procedure resulted in 245 g of crude aqueous extract (AE), corresponding to a 12.25% extraction yield.

Alternatively, 2000 g of powder was macerated for 72 h in 6 L of water‐ethanol mixture (20/80). The resulting solution was filtered using Whatman No. 4 paper. The filtrates obtained were concentrated in a rotary evaporator under reduced pressure (337 mbar at 40°C) and dried in an oven for 24 h at 50°C. A total of 272 g of crude hydro‐ethanolic extract (EE) was obtained, corresponding to a yield of 13.6%, and stored at 4°C.

#### 2.2.2 Determination of Secondary Metabolite Classes

The phytochemical profile of aqueous (AE) and ethanolic (EE) extracts was evaluated by quantifying the major classes of secondary metabolites. The total phenolic content was estimated using the Folin–Ciocalteu colorimetric assay [14]. Tannin contents were assessed through the acidified vanillin spectrophotometric method, as outlined in previous reports [15]. Flavonoid concentration was measured in accordance with the method developed by Chang et al. [16], whereas total alkaloid content was measured in compliance with the protocol revised by Singh et al. [17] Total saponin content was determined using the vanillin‐sulfuric acid protocol [18].

#### 2.2.3 In Vitro Assessment of Antioxidant Activity

The free radical scavenging potential activity of *Erythrina excelsa* extracts was determined using the 2,2‐diphenyl‐1‐picrylhydrazyl (DPPH) assay, following the procedure described by Sanchez‐Moreno et al. [19], with minor modifications. The ferric reducing antioxidant power (FRAP) was quantified as indicated by Re et al. [20].

### 2.3 Cell Based Assay

#### 2.3.1 Cell Lines and Cell Cultures

Human triple negative basal cell line (MDA‐MB‐231), estrogen sensitive cell line (MCF‐7), and Human Mammary Epithelial Cells (HMEC) deriving from normal mammary cells were supplied by American Type Culture Collection (ATCC/LGC) Promochem (Wesel, Germany).

Cells were grown and subcultured in RPMI‐1640 media supplemented with 10% fetal calf serum (FCS) and 1% penicillin (100 U/mL)/streptomycin (100 μg/mL). They were incubated in a humid 5% CO_2_ incubator maintained at 37°C and pH 7.4 pH. For cell passage, 90% of the supernatant was replaced with fresh medium every 2 days. Before each experiment, the number of viable cells was estimated by the trypan blue exclusion test through the BioRad equipment.

#### 2.3.2 Cell Growth Assessment

The growth of cells was assessed using the 3‐(4,5‐dimethylthiazol‐2‐yl)‐2,5‐diphenyl tetrazolium bromide (MTT) assay. The hormone‐sensitive (MCF‐7) and triple‐negative (MDA‐MB‐231) breast cancer cells were seeded at a density of 7 × 10^3^ cells per well in 96‐well plates containing 100 μL of complete growth medium and incubated for 24 h at 37°C in a humidified atmosphere with 5% CO_2_. Cells were then treated with AE and EE at concentrations 12.5, 25, 50, 100, and 200 μg/mL for 24 h under the same incubation conditions. Following treatment, 20 μL of MTT solution (5 mg/mL in 1× PBS) was added to each well, and the plates were incubated for an additional 4 h to allow for formazan crystal formation. The supernatant was carefully removed, and the resulting formazan crystals were solubilized with 100 μL of dimethyl sulfoxide (DMSO). Absorbance was measured at 570 nm using a microplate ELISA reader. Cell viability was expressed as a percentage relative to untreated controls, and the percentage of growth inhibition was calculated using the formula: % of inhibition of cell proliferation = 1‐[(OD_(570nm)_ test/OD_(570nm)_ control)] × 100.

#### 2.3.3 Flow Cytometry to Determine Cell Cycle Stage

Exponentially growing cells (2 × 10^6^) were treated with AE and EE at its IC_50_ concentration for 24 h. Thereafter, the cells were harvested, washed with cold PBS, and fixed in chilled acetone for 20 min. The fixed cell suspension was then subjected to centrifugation (5000 rpm, 3 min). Then, residual acetone was removed, and the pellet was washed with PBS and re‐centrifuged. The cells were resuspended in 5 μL of RNase solution (10 mg/mL), incubated at 37°C for 25 min, and stained with 10 μL of propidium iodide (PI, 10 μg/mL) followed by a 30‐min dark incubation. The samples were then analyzed using a FACScan flow cytometer (Beckton Dickinson), with fluorescence detected on the FL2H channel (logarithmic amplification). Finally, cell cycle distribution was determined by counting 10 000 cells per sample.

#### 2.3.4 Acridine Orange /Propidium Iodide Co‐Staining Assay

The acridine orange (AO)/propidium iodide (PI) double staining assay was performed following established fluorescence microscopy protocols using a Leica system equipped with Q‐Floro software. MCF‐7 and MDA MB 231 cells, cultured in 25 mL Nunc flasks, were treated with AE at its IC_50_ for 24 h. Post‐treatment, cells were harvested, washed with PBS, and stained by adding 10 μL each of AO/PI solutions (10 μg/mL) to the cell pellet. After gentle mixing, samples were incubated for 5 min at room temperature in the dark. Morphological changes indicative of apoptosis or necrosis were immediately assessed (within 30 min to prevent fluorescence fading) using an Olympus BX51 fluorescence microscope. Images were captured in AO (green fluorescence; viable/apoptotic cells) and PI (red fluorescence; necrotic cells) channels, with analysis conducted via Q‐Floro software to quantify fluorescence signals and cellular integrity.

### 2.4 In Vivo Chemopreventive Experimental Design

The in vivo experimentation focused exclusively on the aqueous extract (AE) of *Erythrina excelsa*, selected because it is the one empirically used by the traditional healer and for its quasi‐similar effect with ethanolic (EE) extract on in vitro antioxidant activity, as well as its pronounced effects on growth inhibition, cell cycle modulation, and apoptosis induction in the MCF‐7 and MDA‐MB‐231 breast cancer cell lines.

#### 2.4.1 Animals

A total of forty‐two (42) female Wistar rats, aged 45–55 days old and weighing approximately 80 g at the beginning of the experiment, were obtained from the Animal Physiology and Pharmacology Laboratory of the University of Maroua, Cameroon. The animals were housed in standard plastic cages under controlled conditions (25°C ± 2°C; 12 h light/12 h dark cycle). Rats were provided unrestricted access to drinking water and were maintained on a soy‐free diet composed of maize meal (50%), fish meal (15%), palm oil (0.1%), bone meal (4%), cottonseed meal (10%), table salt (0.8%), and a vitamin complex (olititiazol, 0.02%).

All procedures for animal housing, handling, and experimentation complied with the European Union Directive 2010/63/EU on the protection of animals used for scientific purposes. This study protocol has been approved by the National Committee for Institutional Ethics of Cameroon under the Ministry of Scientific Research and Technological Innovation (Registration No. FWA‐IRD 0001954).

#### 2.4.2 Dose Determination

To establish an experimental model of mammary carcinogenesis, female prepubertal rats (aged 45–55 days) were administered 7,12‐dimethylbenz(a)anthracene (DMBA), a well‐known carcinogen. DMBA was prepared at a dose of 50 mg/kg BW, in 0.3 mL of olive oil and delivered via subcutaneous injection into the mammary region. The control animals received an equivalent volume of olive oil alone [21]. Tamoxifen, a SERM, was used at 3.3 mg/kg BW as the standard reference treatment [22]. Experimental groups were assigned to receive aqueous extract of *E. excelsa* at doses of 75, 150 and 300 mg/kg BW in order to evaluate dose‐dependent effects.

#### 2.4.3 DMBA Induces a Mammary Tumor in the Female Rat Model

Following a 10‐day acclimatization period, the rats were randomized to six experimental groups (*n* = 7 per group). Animals in the normal group received 0.3 mL of olive oil subcutaneously, whereas all other groups were administered DMBA (50 mg/kg BW) dissolved in 0.3 mL of olive oil for eight consecutive days. Post‐induction, the groups were treated as follows: Group 1 served as the normal control (NOR) and group 2 as the negative control (DMBA) both received a vehicle (2% ethanol in distilled water); Group 3 (positive control), was treated with tamoxifen (3.3 mg/kg BW); and Groups 4–6 received AE at doses of 75, 150 and 300 mg/kg BW, respectively. All treatments were administered once daily via intragastric gavage for a period of 140 days.

Animals were treated with amoxicillin by oral gavage for 10 days immediately after exposure to DMBA, to minimize discomfort and prevent bacterial infections. Weight was recorded on a weekly basis and mammary glands were palpated twice weekly to detect tumor development. At the end, of experimental period, animals were anesthetized with ketamine (50 mg/kg BW) and diazepam (10 mg/kg BW), followed by euthanasia via cervical dislocation. Blood samples were collected after the sacrifice in EDTA‐coated tubes for hematological analysis, whereas serum for biochemical analyses was obtained by centrifuging samples in dry tubes at 3000 rpm for 15 min at 4°C for biochemical analysis. Mammary tumors were excised, weighed and their dimensions measured with a precision caliper (1 mm; IGAGING, Nice, France). Tumor volume was calculated using the formula proposed by Faustino‐Rocha et al. [23]. Tumors were then homogenized on ice in 0.1 M sodium phosphate buffer (pH 7.5) to yield a 20% homogenate. Estrogen‐sensitive organs (uterus, vagina, mammary glands), major sites of breast cancer metastasis (brain, lungs, liver), and other toxicity‐related organs (spleen, kidney, adrenal gland), were also removed, counted, weighed, and fixed in 10% neutral formalin for histopathological and biochemical analysis.

#### 2.4.4 Histological Analysis

The tissue samples were processed using standard histological techniques, which included tissue trimming, dehydration, and paraffin embedding. Serial sections with a thickness of 5 μm were prepared from organs embedded in paraffin. The sections were then stained with the hematoxylin–eosin (H&E) method and mounted with resin for microscopic examination. The microarchitecture of tumors and selected organs was assessed using an Axioskop 40 light microscope equipped with a Celestro digital camera. Quantitative and qualitative analysis of histological images was performed using the Image J software [24].

#### 2.4.5 Evaluation of Biochemical Parameters

Serum total protein levels were quantified using the Biuret method as outlined by Gornall et al. [25]. Malondialdehyde (MDA), superoxide dismutase (SOD) and catalase activities were evaluated following the protocols described by Wilbur et al. [26], Misra and Fridovich [27] and Dimo et al. [28], respectively.

Pro‐inflammatory and angiogenic cytokines, including tumor necrosis factor‐alpha (TNF‐α), interferon‐gamma (IFN‐γ), interleukins 6 (IL‐6), 12 (IL‐12), and vascular endothelial growth factor (VEGF) as well as breast associated biomarker CA15‐3, were measured in serum using enzyme‐linked immunosorbent assays (ELISA), as previously reported (Fotsing et al. [24]).

The systemic effects of EE were further evaluated by determining hepatic, renal, and lipid profiles. Serum levels of aspartate aminotransferase (AST), alanine aminotransferase (ALT), urea and creatinine, triglycerides, and cholesterol were analyzed using commercially available colorimetric assay kits (Bio‐diagnostic Co., Giza, Egypt) according to the manufacturer's instructions.

### 2.5 Statistical Analysis

All quantitative data are presented as mean ± and standard error of the mean (SEM). Statistical analyses were conducted using GraphPad Prism 8.01 software version. The analysis was performed in a blinded manner to minimize interpretation bias. Differences among experimental groups were evaluated using one‐way analysis of variance (ANOVA) followed by Dunnett's multiple comparisons post hoc test. Each in vitro experiment was performed in triplicate across a minimum of three independent experimental runs. Significance was set at *p* < 0.05.

## 3 Results

### 3.1 Phytochemical Analysis and In Vitro Antioxidant Potential

Phytochemical analysis of the aqueous (AE) and ethanolic (EE) extracts of *Erythrina excelsa* revealed the presence of several classes of secondary metabolites, including flavonoids, flavonols, phenols, and tannins. Quantitative assessment indicated that polyphenols were the most abundant constituents (348.78 ± 2.77 mg EAA/g DW in AE and 434.22 ± 3.56 mg EAA/g DW in EE), followed by flavonoids (31.94 ± 0.96 mg quercetin equivalents/g DW in AE and 39.35 ± 1.22 mg quercetin equivalents/g DW in EE), while tannins (9.98 ± 0.37 mg EAT/g DW in AE and 12.71 ± 0.42 mg EAT/g DW in EE) and flavonols (7.35 ± 0.09 mg quercetin equivalents/g DW in AE and 9.53 ± 0.04 mg quercetin equivalents/g DW in EE) were detected in lower amounts (Table 1A).

**TABLE 1:** Quantitative phytochemical composition and antioxidant activities of *E. excelsa* aqueous (AE) and ethanolic (EE) extract.

Metabolites (A)	Aqueous extract (AE)	Ethanolic extract (EE)
Total phenols (mg EAA/g DW)	348.78 ± 2.77	434.22 ± 3.56
Flavonoids (μg eq/g DW)	31.94 ± 0.96	39.35 ± 1.22
Flavonols (μg eq/g DW)	7.35 ± 0.09	9.53 ± 0.04
Tannins (mg ETA/g DW)	9.98 ± 0.37	12.71 ± 0.42
*Anti‐oxidant activity* (*B*)		
FRAP (IC_50_ in μg/mL)	4765.34 ± 26.45	5135.12 ± 19.24
DPPH (IC_50_ in μg/mL)	19.72 ± 35.18	17.23 ± 13.11

*Note:* Results are expressed as mean ± standard deviation (SD) of three independent experiments. IC_50_ values represent the extract concentration required to achieve 50% radical scavenging activity (DPPH) or ferric reduction (FRAP).

Abbreviations: DW, dry weight; EAA, equivalent ascorbic acid; ETA, equivalent tannic acid.

The antioxidant potential of AE and EE was further evaluated using the DPPH radical scavenging assay and the ferric reducing antioxidant power (FRAP) assay. The extracts displayed significant free radical scavenging activity with an IC_50_ value of 19.72 ± 35.18 μg/mL and 17.23 ± 13.11 μg/mL in the DPPH assay for AE and EE extracts, respectively. In addition, these extracts demonstrated considerable reducing capacity, converting Fe^3+^ to Fe^2+^ at a rate of approximately 75%, corresponding to an IC_50_ value of 4765 ± 26.45 μg/mL for AE and 5135.12 ± 19.24 μg/mL for EE (Table 1B).

### 3.2 Cell Growth and Cell Proliferation

#### 3.2.1 Cell Growth

**FIGURE 1:**
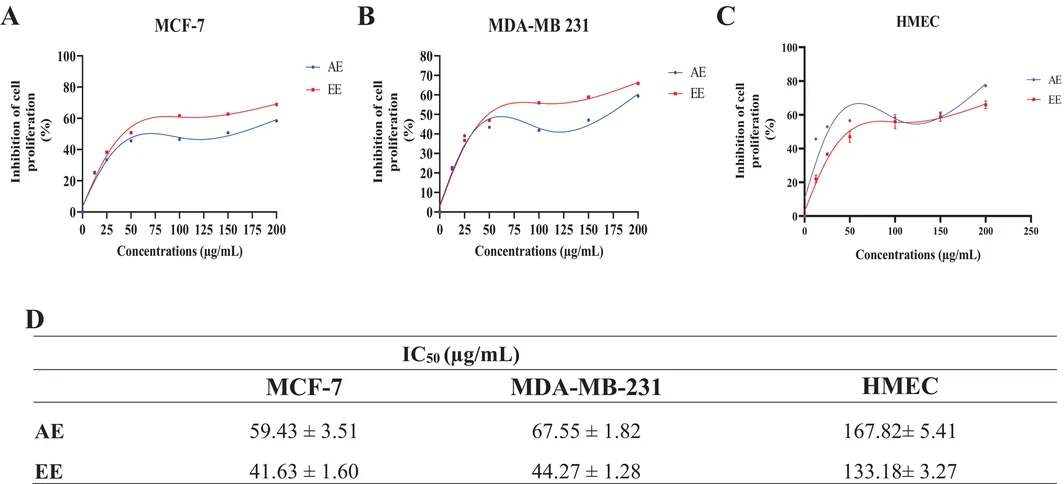
Growth of estrogen‐sensitive MCF‐7 (A) and non‐sensitive MDA‐MB 231 (B) breast carcinoma cells as well as non‐cancerous HMEC (C) treated with different concentrations of *E. excelsa* extracts after 24 h and IC_50_ (D). Treated cancer cell cultures were compared to untreated control cultures of the same passage and the same number of cells per well.

The growth of MCF‐7 and MDA‐MB 231 cells was inhibited in a dose‐dependent manner 24 h post treatment. Aqueous extract of *Erythrina excelsa* (AE) exhibited cytotoxic effect with half‐maximal inhibitory concentration (IC_50_) values of 59.43 ± 3.51 μg/mL (MCF‐7 cells), 67.55 ± 1.82 μg/mL (MDA‐MB‐231 cells), and 167.82 ± 5.41 μg/mL (HMEC cells). However, EE exhibited the most prominent effect with an IC_50_ value of 41.63 ± 1.60, 44.27 ± 1.28 μg/mL, and 148.75 ± 6.33 μg/mL for MCF‐7, MDA‐MB 231, and HMEC cells, respectively (Figure 1). The selectivity index was > 2.5, suggesting that *E. excelsa* extracts are more effective against cancer cells than non‐cancerous cells.

#### 3.2.2 Cell Cycle Arrest

**FIGURE 2:**
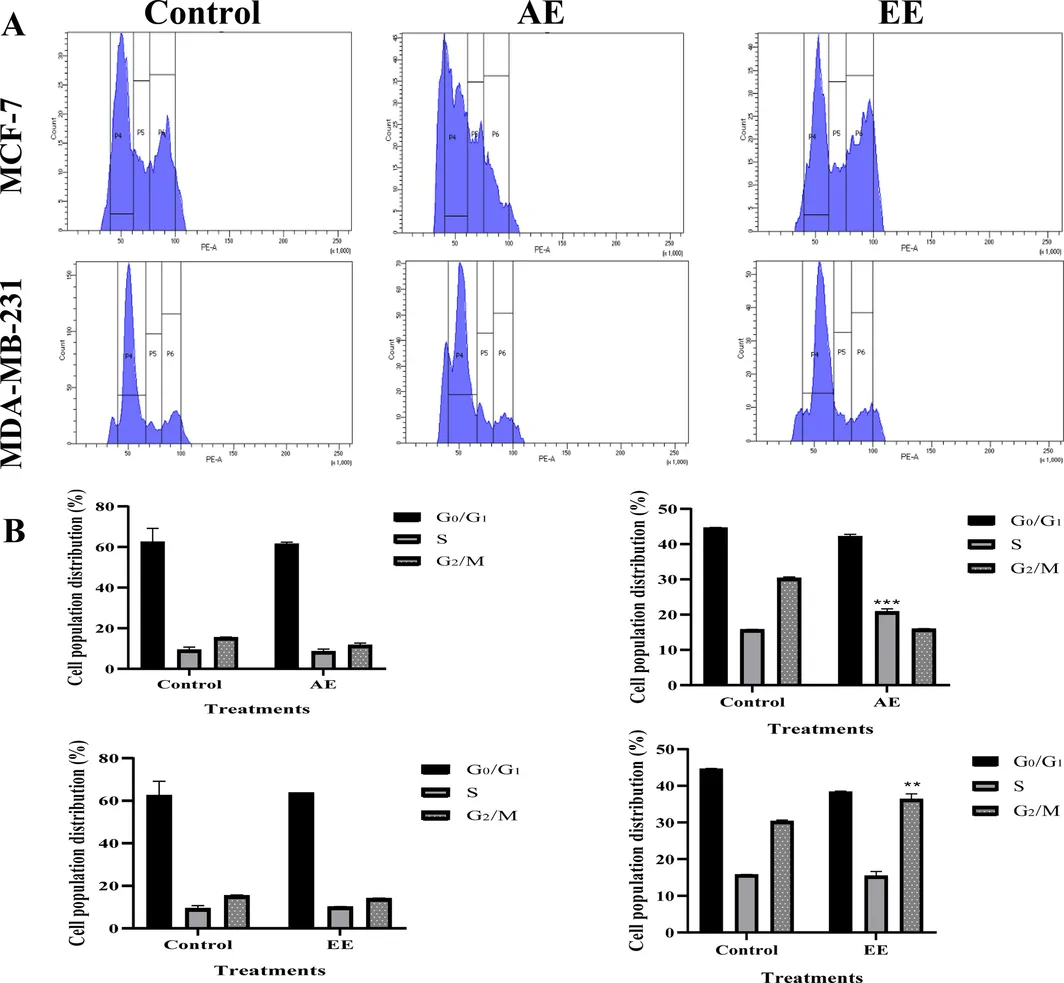
Flow cytometric results: Dot plots (A) and graphic representation (B) of the distribution of the MCF‐7 and MDA‐MB 231 cells exposed to AE and EE at the various stages of the cell cycle. Values obtained as Mean ± SD of triplicate experimental set up. *p < 0.01; **p < 0.001 compared to control after 24 h.

The effects of *Erythrina excelsa* extracts on cell cycle progression in breast cancer cells were evaluated by flow cytometry to elucidate potential mechanisms underlying growth inhibition and cell death (Figure 2). Treatment with the aqueous extract (AE) induced a marked accumulation of MCF‐7 cells in the S phase, increasing the cell population from 15.75% in controls to 20.85% after 24 h of exposure. Meanwhile, the ethanolic extract (EE) caused a significant G2/M phase arrest, with the proportion of MCF‐7 cells rising from 30.35% in controls to 36.4% following 24 h treatment. However, neither extract produced a statistically significant alteration in the cell cycle distribution of MDA‐MB‐231 cells under the same conditions.

#### 3.2.3 Mechanism of Cell Death (Apoptosis or Necrosis)

**FIGURE 3:**
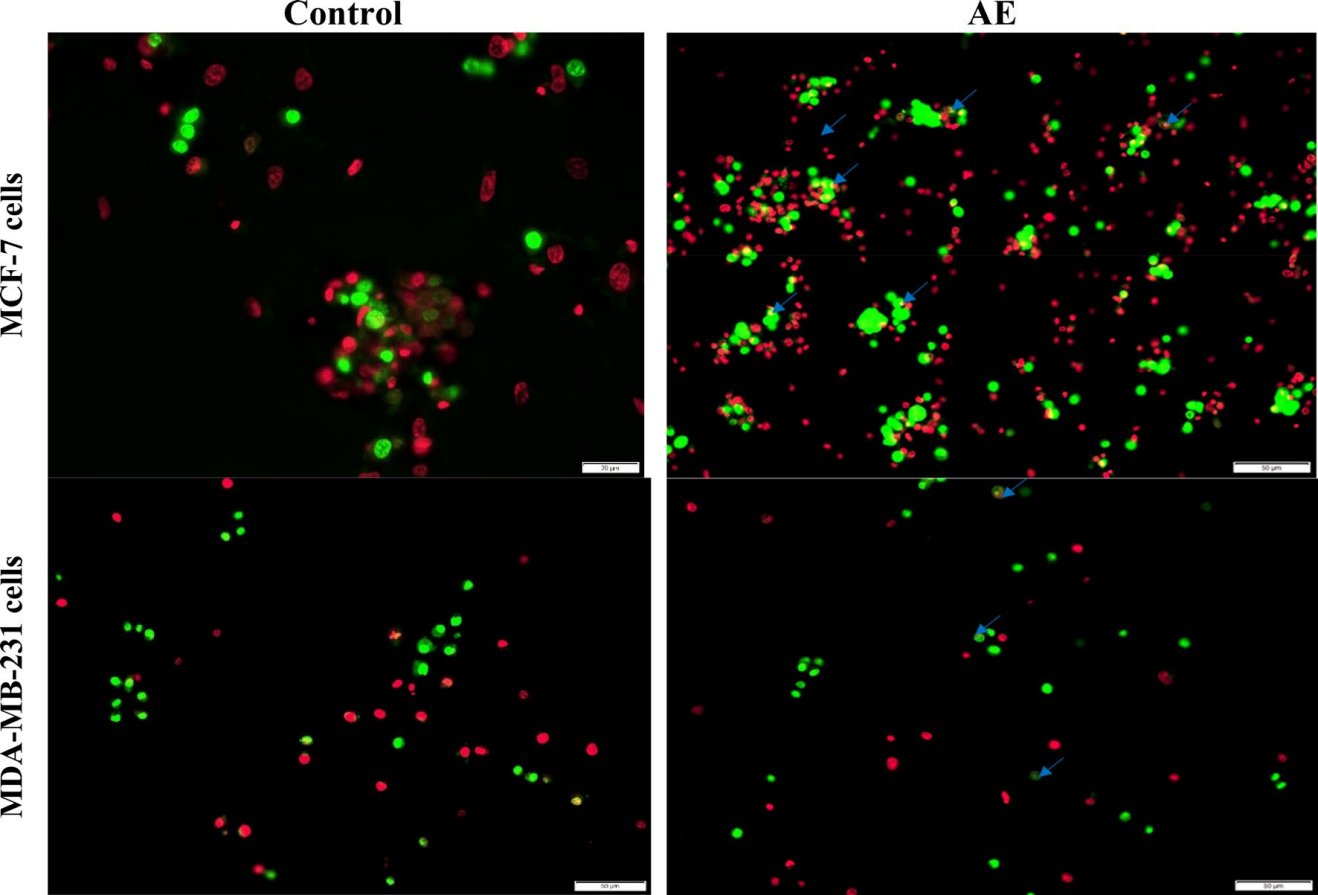
Micrographs of acridine orange (AO) and propidium iodide (PI) double‐stained against breast cancer cells untreated and treated MCF‐7 and MDA MB 231 cells with AE at IC_50_ concentration for 24 h. The presence of reddish‐orange staining cells upon treatment indicates the apoptotic features such as chromatin condensation, membrane cell blebbing, and apoptotic bodies. (magnification: 400×).

The apoptotic effect of AE on breast cancer cells was assessed through morphological observation using AO/PI double staining under a fluorescence microscope. The morphological changes were examined in MCF‐7 and MDA‐MB‐231 cells following treatment with extracts at IC_50_ for 24 h (Figure 3). In both MCF‐7 and MDA‐MB‐231 cell lines, untreated cells displayed typical healthy morphology with uniform green fluorescence, indicating intact nuclei and viable cells. Upon AE treatment, numerous cells exhibited hallmark features of apoptosis, including cell membrane blebbing, chromatin condensation, and the formation of apoptotic bodies, as evidenced by reddish‐orange fluorescence. The increase in orange‐red stained cells following treatment suggests a progression to late‐stage apoptosis, confirming that AE induced apoptotic cell death in both breast cancer cell types.

### 3.3 In Vivo Chemo‐Preventive Effects

#### 3.3.1 Effect on Body Weight

**FIGURE 4:**
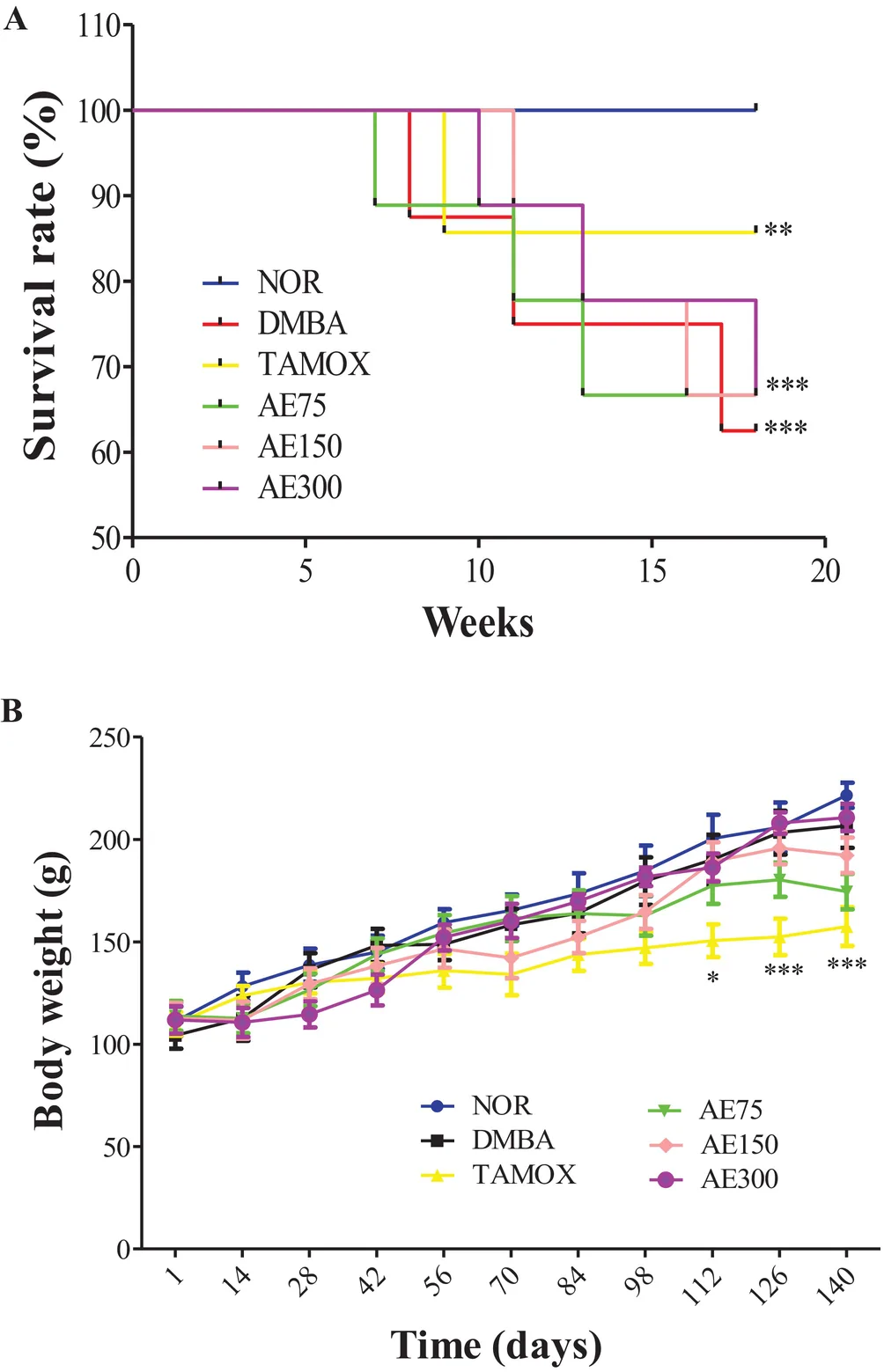
Effects of AE on body weight (A) and survival rate (B). The normal (NOR) and negative control (DMBA) groups received 2% ethanol (vehicle); Tamox + DMBA = positive control rats received tamoxifen as standard at 3.3 mg/kg BW; AE + DMBA = rats treated with the aqueous extract of the bark of *Erythrina excelsa* at doses of 75, 150 and 300 mg/kg body weight, respectively. The values represent the means ± SEM (*n* = 8). All rats, except normal animals, were administered DMBA at a dose of 50 mg/kg. Significance in relation to the NOR group: Significance in relation to the DMBA group: *p < 0.05, **p < 0.01, ***p < 0.001.

Changes in body weight and survival are presented in Figure 4. No mortality was observed in the normal control (NOR) group, while survival in the DMBA group was 60%.

Treatment with AE at 75, 150 and 300 mg/kg resulted in a survival rate of 66.7%, whereas the tamoxifen group showed 87.5% survival (Figure 4A). A progressive decrease in body weight was observed in the tamoxifen group from day 56 to the end of the experimental period, although significant only from day 112 (*p* < 0.05) to 140 (*p* < 0.01). No significant differences in body weight were recorded between the AE‐treated groups and the NOR group (Figure 4B).

#### 3.3.2 Effect on Selected Tumor Parameters

As shown in Table 2, all DMBA‐treated rats (100%) developed mammary tumors, while no atypical acini were observed in the NOR group. Tamoxifen reduced tumor incidence to 37.5% and significantly decreased tumor mass and tumor burden (*p* < 0.001), achieving a 44% inhibition of tumor burden compared with DMBA. AE treatment at all doses reduced tumor incidence, tumor mass, and tumor burden significantly (*p* < 0.001), with tumor burden inhibition exceeding 85%.

**TABLE 2:** Effects of *E. excelsa* aqueous extract on tumor incidence, burden and inhibition percentage.

	Number of rats with tumors	Tumor incidence	Tumoral mass (mg/kg)	Tumor burden (g/kg)	Tumor burden inhibition
NOR	0/8	0%	—	—	—
DMBA	8/8	100%	20.28 ± 4.25	96.17 ± 17.17	—
TAMOX	3/8	37.5%	7.09 ± 2.18***	51.01 ± 1.32**	44%
AE 75	5/8	62.5%	2.63 ± 1.01***	16.79 ± 6.01***	85%
AE 150	2/8	25%	0.25 ± 0.05***	0.92 ± 0.37***	99%
AE 300	4/8	50%	1.69 ± 0.36***	8.81 ± 1.88***	92%

*Note:* The normal (NOR) and negative control (DMBA) groups received 5% ethanol (vehicle); Tamox + DMBA = positive control rats received tamoxifen as standard at 3.3 mg/kg bw; AE + DMBA = rats treated with the aqueous extract of the bark of *Erythrina excelsa* at doses of 75, 150 and 300 mg/kg body weight, respectively. The values represent the means ± SEM (*n* = 8). All rats, except normal animals, were administered DMBA at a dose of 50 mg/kg. Significance in relation to the NOR group. ###*p <* 0.001. Significance in relation to the DMBA group: **p < 0.01, ***p < 0.001.

#### 3.3.3 Effects on Tumor Volume and CA15‐3 Level

**FIGURE 5:**
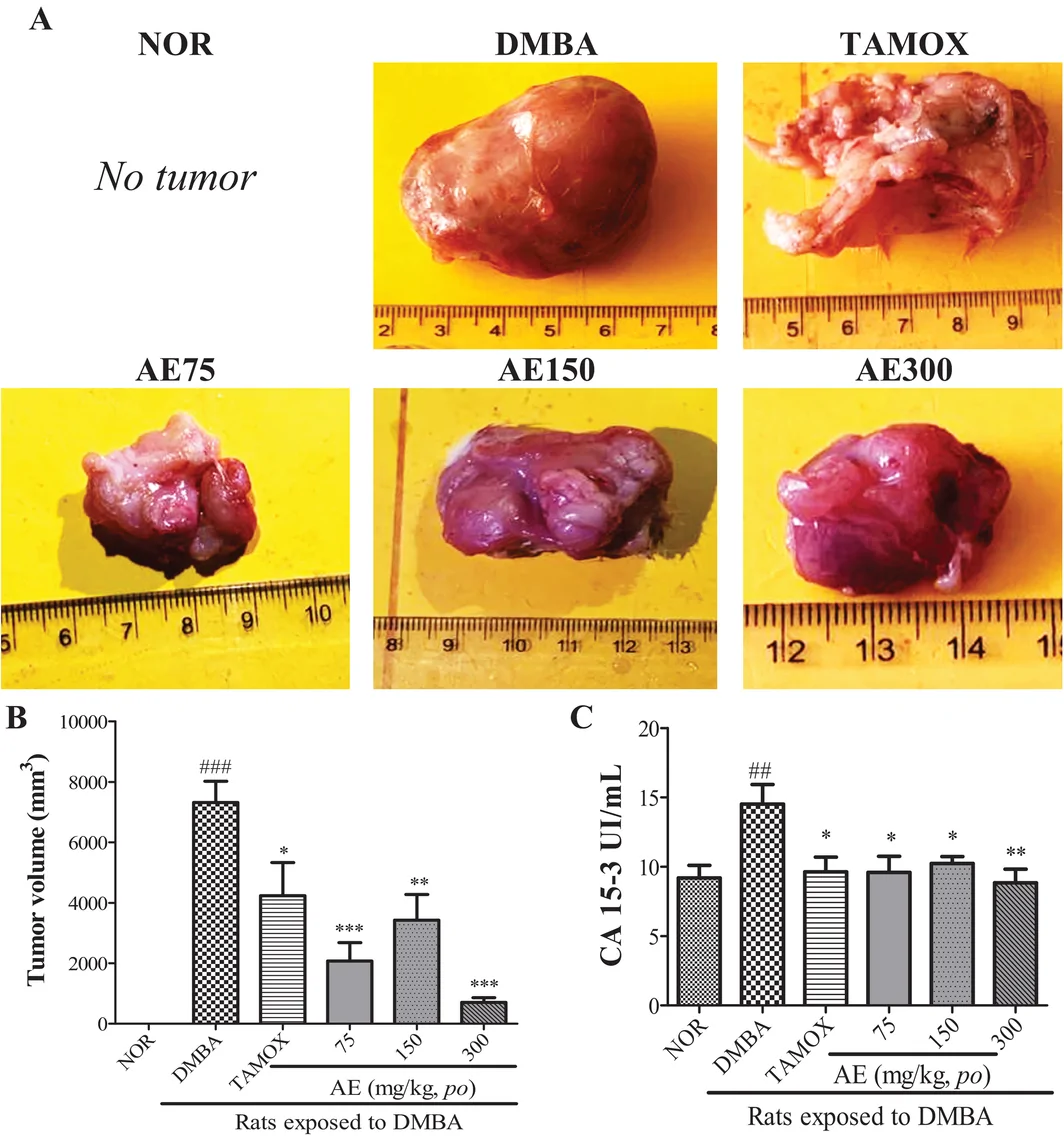
Effects of AE on tumor morphology (A), tumor volume (B), and CA15‐3 level (C). The normal (NOR) and negative control (DMBA) groups received 2% ethanol (vehicle); Tamox + DMBA = positive control rats received tamoxifen as standard at 3.3 mg/kg bw; AE + DMBA = rats treated with the aqueous extract of the bark of *Erythrina excelsa* at doses of 75, 150 and 300 mg/kg body weight, respectively. The values represent the means ± SEM (*n* = 8). All rats, except normal animals, were administered DMBA at a dose of 50 mg/kg. Significance in relation to the NOR group: ##*p <* 0.001 and ###*p <* 0.001. Significance in relation to the DMBA group: *p < 0.05, **p < 0.01, ***p < 0.001.

Rats in the DMBA group developed the largest mammary tumors and exhibited significantly elevated serum CA15‐3 levels (*p* < 0.01) compared with NOR animals (Figure 5A,B). Tamoxifen treatment significantly (*p* < 0.05) reduced the tumor volume and serum CA15‐3 level compared to the DMBA group. Treatment with AE at all doses produced comparable effects to tamoxifen, with significant reductions in serum CA15‐3 levels (*p* < 0.05) and tumor volume (*p* < 0.01) at all doses tested.

#### 3.3.4 Effects on Mammary Glands and Tumor Architecture

**FIGURE 6:**
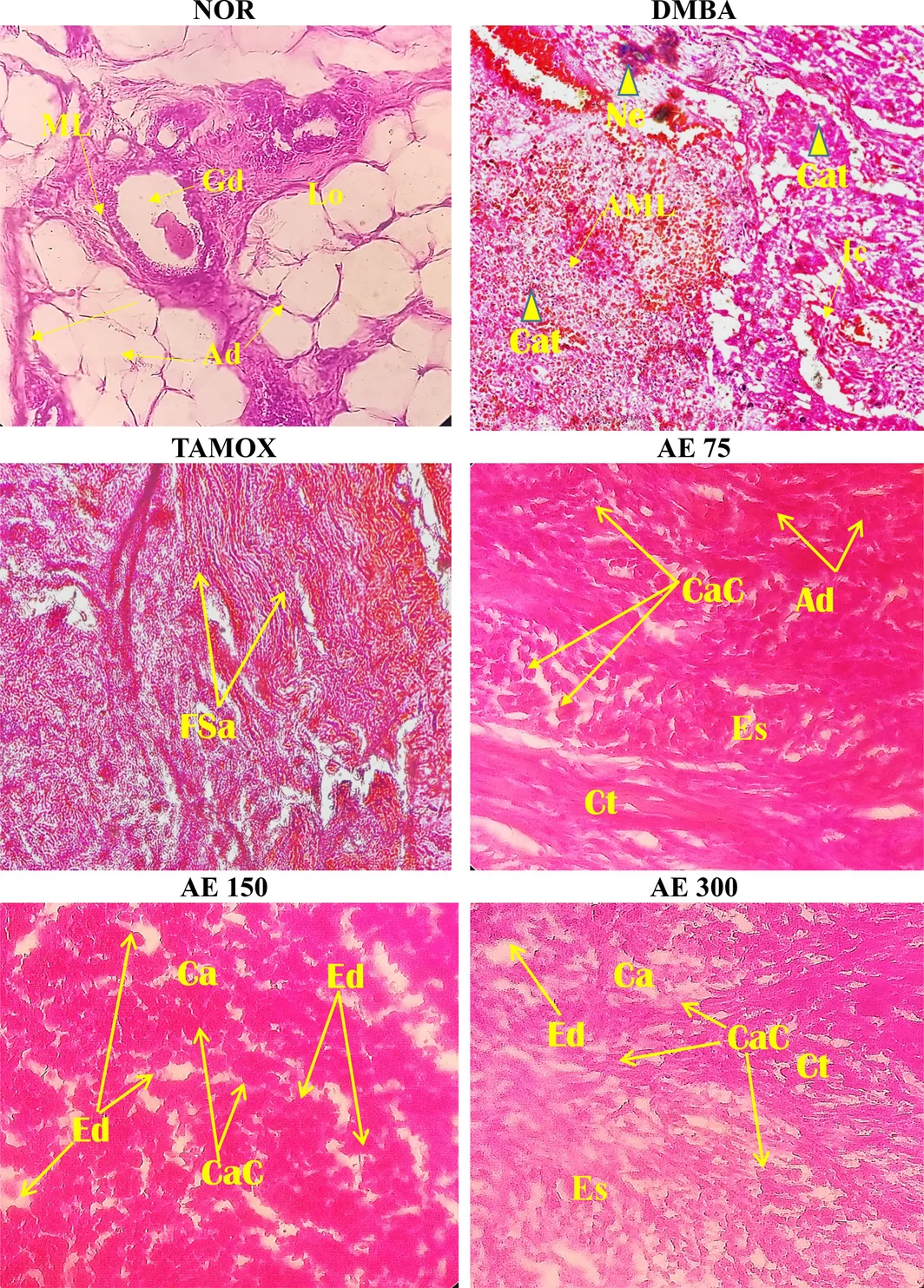
Histopathology of mammary glands and mammary tumours after treatment with aqueous extract of *Erythrina excelsa* (AE) bark. The normal (NOR) and negative control (DMBA) groups received 5% ethanol (vehicle); Tamox + DMBA = positive control rats received tamoxifen as standard at 3.3 mg/kg bw; AE + DMBA = rats treated with the aqueous extract of the bark of *Erythrina excelsa* at doses of 75, 150 and 300 mg/kg body weight, respectively. All rats, except normal animals, were administered DMBA at a dose of 50 mg/kg. Ca, carcinoma; CaC, cancerous cells; Ct, conjunctive tissue; Ed, edema; Es, edematous stroma; Gd, galactophoric duct; ML, myoepithelial layers.

Histological analyses after 20 weeks revealed normal mammary architecture in the NOR group, characterized by adipocyte lobules separated by connective tissue. Conversely, DMBA‐exposed rats displayed marked architectural alterations, including connective tissue hyperplasia, ductal epithelial hyperplasia with loss of myoepithelial layers, spindle‐cell proliferation forming intersecting bundles, multinucleated giant cell infiltration, and extensive necrosis. Tamoxifen‐treated rats developed invasive lobular carcinoma with clustered tumor proliferation. The AE treatment modified tumor architecture in a dose‐dependent manner. At 75 mg/kg, tumors were characterized by spindle‐cell proliferation arranged in sheets. At 150 mg/kg, tumors consisted of trabeculae‐like arrangements of non‐cohesive tumor cells infiltrating edematous stroma. At 300 mg/kg, only rare, loose tumor cell plaques persisted within necrotic areas (Figure 6).

#### 3.3.5 Oxidative Stress and Protein Levels in Mammary Tissue

**FIGURE 7:**
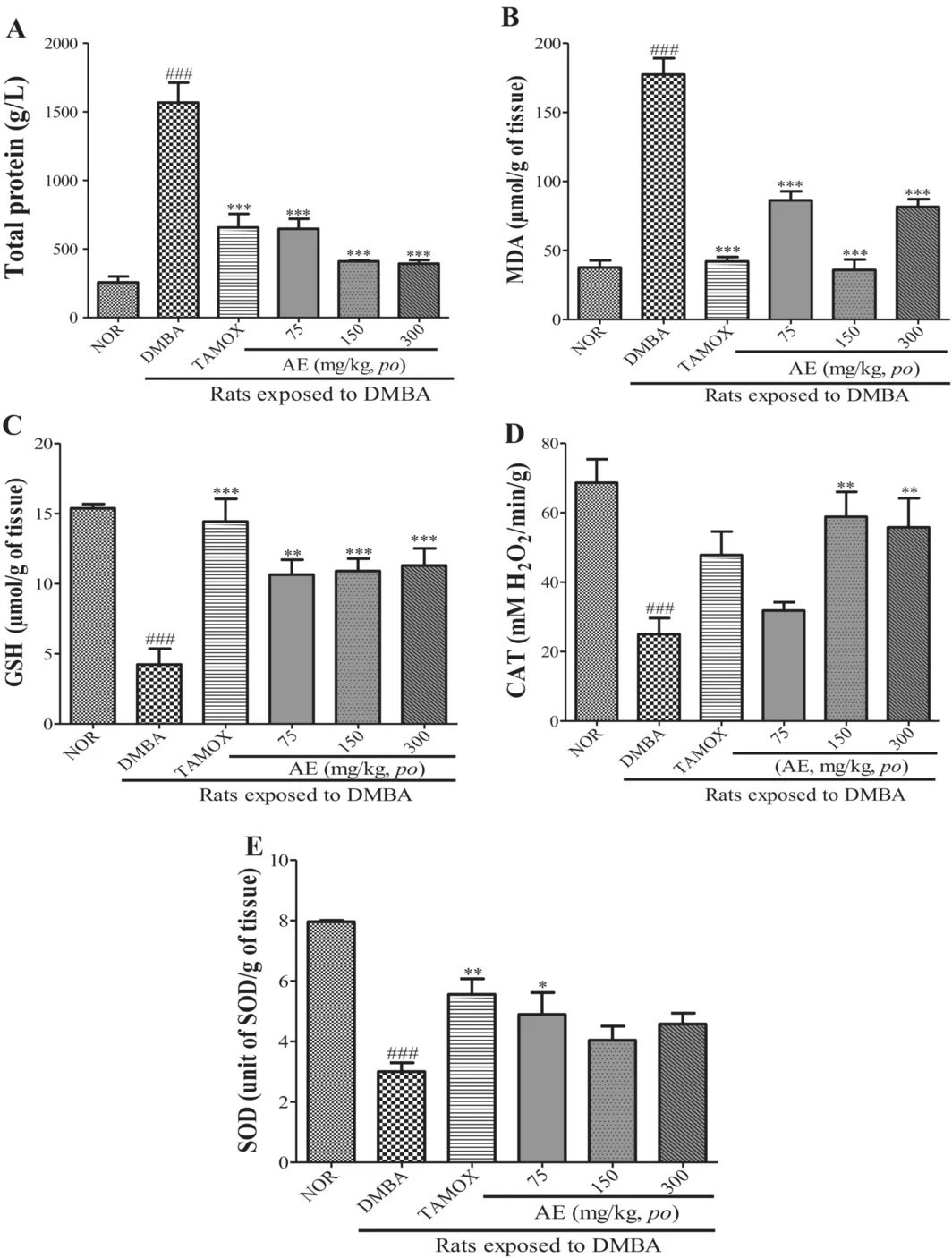
Effects of AE on Total protein (A), MDA level (B) and GSH (C) levels as well as catalase (D) and SOD (E) activities. The normal (NOR) and negative control (DMBA) groups received 5% ethanol (vehicle); Tamox + DMBA = positive control rats received tamoxifen as standard at 3.3 mg/kg BW; AE + DMBA = rats treated with the aqueous extract of the bark of *Erythrina excelsa* at doses of 75, 150 and 300 mg/kg body weight, respectively. The values represent the means ± SEM (*n* = 8). All rats, except normal animals, were administered DMBA at a dose of 50 mg/kg. Significance in relation to the NOR group: ###*p <* 0.001. Significance in relation to the DMBA group: *p < 0.05, **p < 0.01, ***p < 0.001.

The effects of AE on oxidative stress parameters and total proteins is shown in Figure 7. Total protein levels were significantly increased in the DMBA group (*p* < 0.001). This was significantly (*p* < 0.001) reduced by tamoxifen and all the tested doses of AE (Figure 7A). DMBA significantly (*p* < 0.001) increased MDA and total protein levels and decreased GSH level as well as SOD and CAT activities compared with the NOR group. Tamoxifen and AE at all tested doses (*p* < 0.001) showed significant prevention of DMBA‐induced lipid membrane peroxidation (Figure 7B).

As expected, rats in the DMBA group had a lower GSH level (*p <* 0.001) compared to normal rats. Meanwhile, a significant increase of GSH level was noted in tamoxifen (*p* < 0.001) and all the AE‐treated (*p* < 0.01 at 75 mg/kg and *p* < 0.001 at 300 mg/kg) compared to the DMBA group (Figure 7C). Catalase activity, decreased in the DMBA group, was increased by tamoxifen (non‐significant) and AE at doses of 150 mg/kg (*p <* 0.01) and 300 mg/kg (*p <* 0.01) (Figure 7D).

SOD activity, reduced in DMBA rats (3.01 ± 0.29 unit of SOD/g of tissue) versus NOR (7.96 ± 0.04 unit of SOD/g of tissue), was significantly restored by tamoxifen (5.56 ± 0.51 unit of SOD/g of tissue; *p* < 0.01) and by AE at 75 (4.89 ± 0.72 unit of SOD/g of tissue; *p* < 0.05) (Figure 7E).

#### 3.3.6 Effects on Some Inflammatory Parameters

**FIGURE 8:**
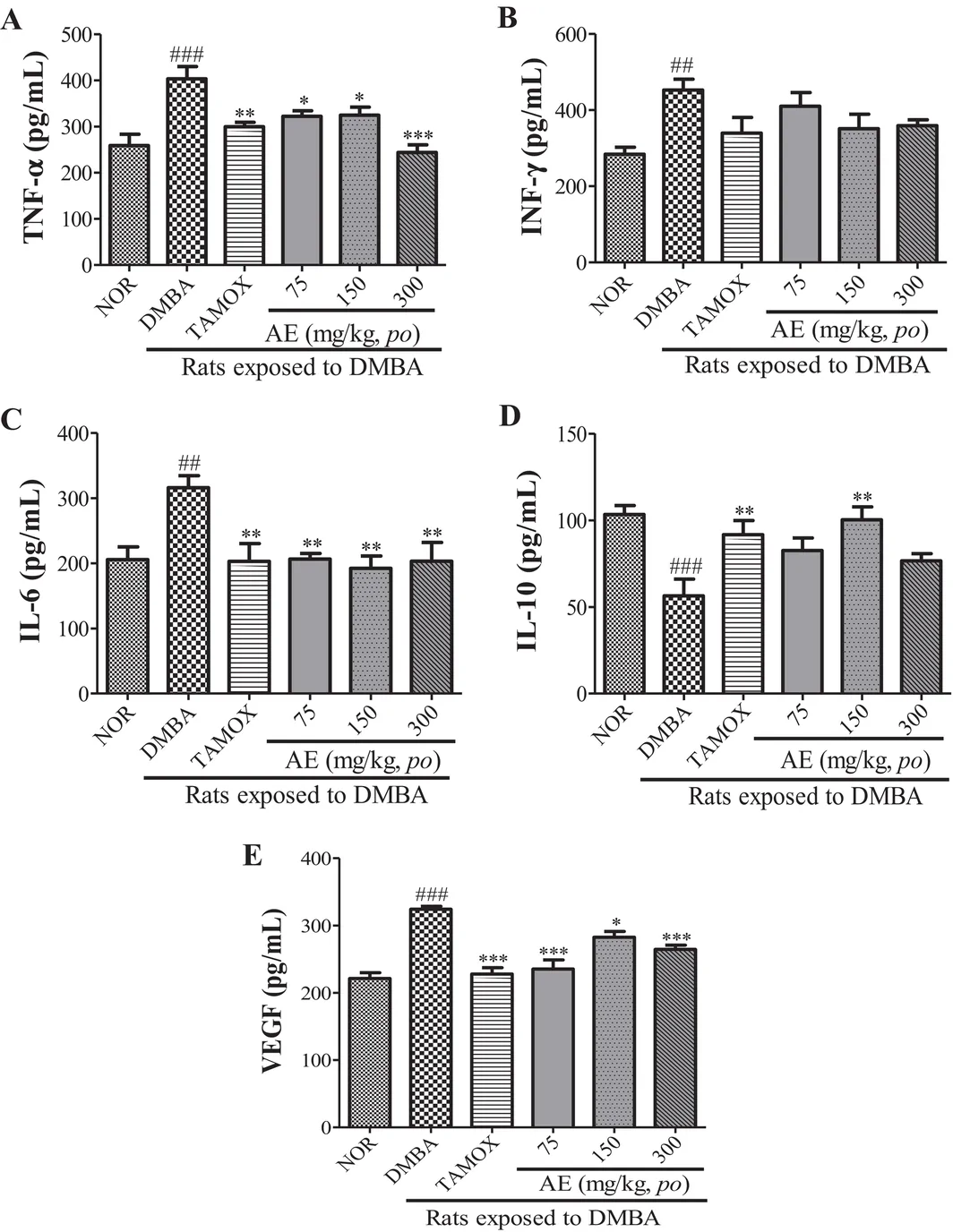
Effects of AE on TNF‐α (A), IFN‐γ (B), IL‐6 (C), IL‐10 (D), and VEGF (E) levels. The normal (NOR) and negative control (DMBA) groups received 2% ethanol (vehicle); Tamox + DMBA = positive control rats received tamoxifen as standard at 3.3 mg/kg BW; AE + DMBA = rats treated with the aqueous extract of the bark of *Erythrina excelsa* at doses of 75, 150, and 300 mg/kg body weight, respectively. The values represent the means ± SEM (*n* = 8). All rats, except normal animals, were administered DMBA at a dose of 50 mg/kg. Significance in relation to the NOR group: ##*p <* 0.01 and ###*p <* 0.001. Significance in relation to the DMBA group: *p < 0.05, **p < 0.01, ***p < 0.001.

Figure 8 illustrates serum cytokine levels after 20 weeks. DMBA significantly increased TNF‐α (*p* < 0.001), IL‐6 (*p* < 0.01), IFN‐γ (*p* < 0.01), and VEGF (*p* < 0.001), while decreasing IL‐10 (*p* < 0.001). Tamoxifen reversed these changes, reducing TNF‐α, IL‐6, VEGF (all *p* < 0.01–0.001), and increasing IL‐10 (*p* < 0.01). AE treatment elicited similar dose‐dependent effects, significantly lowering TNF‐α (at least *p* < 0.05), IL‐6 (*p* < 0.01), and VEGF (at least *p* < 0.05), whereas significantly increasing IL‐10 at 150 mg/kg (*p* < 0.01).

#### 3.3.7 Effects on the Liver, Kidney and Lipidemia Parameters After 20 Weeks of Treatment

**FIGURE 9:**
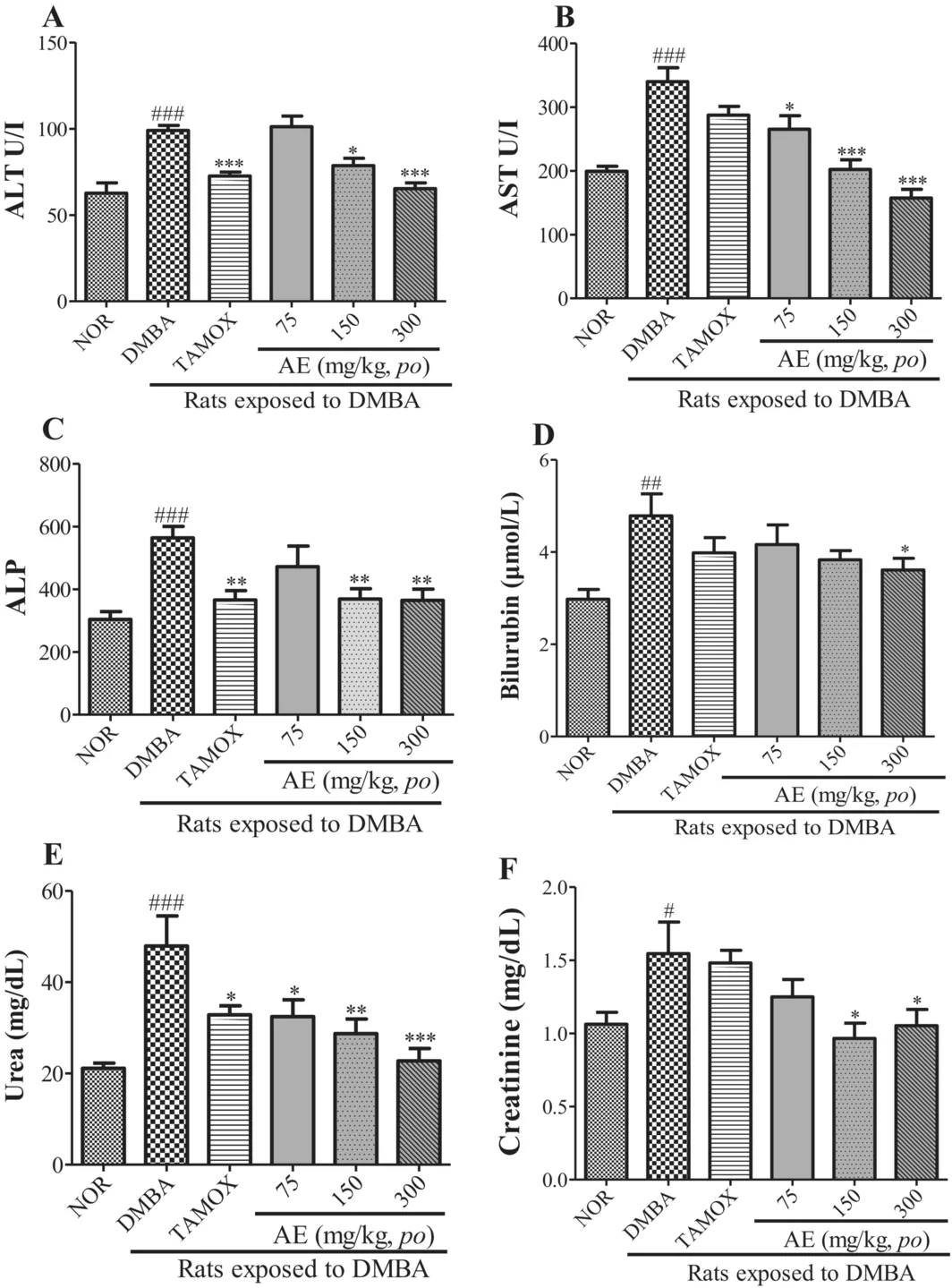
Effects of AE on ALT (A), AST (B), ALP (C), bilirubin (D), urea (E) and creatinine (F) levels. The normal (NOR) and negative control (DMBA) groups received 2% ethanol (vehicle); Tamox + DMBA = positive control rats received tamoxifen as standard at 3.3 mg/kg BW; AE + DMBA = rats treated with the aqueous extract of the bark of *Erythrina excelsa* at doses of 75, 150 and 300 mg/kg body weight, respectively. The values represent the means ± SEM (*n* = 8). All rats, except normal animals, were administered DMBA at a dose of 50 mg/kg. Significance in relation to the NOR group: ##*p <* 0.01 and ###*p <* 0.001. Significance in relation to the DMBA group: *p < 0.05, **p < 0.01, ***p < 0.001.

Biochemical parameters of liver and kidney function are shown in Figure 9. DMBA caused a significant increase in the activity of ALT/AST (*p* < 0.001), alkaline phosphatase (*p* < 0.001), bilirubin level (*p* < 0.01), urea (*p* < 0.001), and creatinine (*p* < 0.05) levels compared with the normal group. Tamoxifen significantly decreased ALT activity (*p* < 0.001), alkaline phosphatase (*p* < 0.01), and urea (*p* < 0.05) levels. Compared to the DMBA group, AE treatment produced significant dose‐dependent reductions in ALT activity at 150 mg/kg (*p* < 0.05) and 300 mg/kg (*p* < 0.001), as well as AST activity at all doses (*p* < 0.05 at 75 mg/kg and *p* < 0.001 at 150 and 300 mg/kg). AE reduced in a dose‐dependent fashion the alkaline phosphatase activity [significant (*p* < 0.01) only at 150 and 300 mg/kg] and bilirubin level (significant (*p* < 0.05) only at 300 mg/kg). At the level of biomarkers of the kidneys function, it significantly counteracted, in a dose‐dependent manner, the DMBA‐induced increase in urea (*p* < 0.05 at 75 mg/kg, *p* < 0.01 at 150 mg/kg, and *p* < 0.001 at 300 mg/kg) and creatinine levels (*p* < 0.05 at 75 mg/kg and 150 mg/kg).

#### 3.3.8 Effects on Hematological Parameters After 20 Weeks of Treatment

Hematological analysis revealed leukocytosis in DMBA rats, with a significant increase in total white blood cells (22.48 ± 0.07 × 10^3^/μL, *p* < 0.001) compared to NOR (8.04 ± 0.5 × 10^3^/μL). This was associated with elevated monocytes (*p* < 0.001) and neutrophils (*p* < 0.001), and a marked decrease in lymphocytes (*p* < 0.001). Treatment with AE led to a significant reduction in WBC counts at 150 and 300 mg/kg (*p* < 0.001), a decrease in granulocyte counts, and a restoration of lymphocyte and monocyte percentages.

DMBA also induced anemia, with significant decreases in RBCs (*p* < 0.001), HCT (*p* < 0.05), and hemoglobin, along with elevated platelet counts (*p* < 0.001). The administration of AE treatment at a dose of 300 mg/kg resulted in a significant increase in HCT (*p* < 0.05) and a reduction in platelet levels (*p* < 0.001) at 150 and 300 mg/kg. In contrast, tamoxifen produced effects that were analogous to those observed in the AE treatment, with similar changes in WBC, granulocytes, HCT, and platelet counts (Table 3).

**TABLE 3:** Effects of *E. excelsa* aqueous extract on some hematological parameters.

	NOR	DMBA	TAMOX	AE 75	AE 150	AE 300
WBC (×10^3^/μL)	8.04 ± 0.5	22.48 ± 0.07###	7.99 ± 1.24***	15.35 ± 1.60	8.75 ± 1.25***	8.56 ± 1.37***
LYMP (×10^3^/μL)	81.30 ± 1.44	34.47 ± 0.36###	77.62 ± 0.92***	50.36 ± 8.90*	70.45 ± 3.73***	45.12 ± 1.45
GRAN (×10^3^/μL)	15.28 ± 1.82	54.75 ± 1.37###	16.25 ± 1.71***	30.82 ± 7.06***	16.52 ± 1.81***	49.86 ± 0.65
MONO (×10^3^/μL)	0.86 ± 0.28	7.45 ± 0.07###	7.93 ± 0.28	10.90 ± 1.26**	14.50 ± 0.60***	5.00 ± 0.70
RBC (×10^6^/μL)	7.14 ± 0.09	5.04 ± 0.17###	6.44 ± 0.80	5.92 ± 0.53	6.53 ± 0.33	7.63 ± 0.33
HGB (g/dL)	13.94 ± 0.12	10.2 ± 0.03^#^	11.78 ± 1.63	12.20 ± 1.57	12.20 ± 0.73	12.52 ± 0.49
HCT (%)	42.19 ± 1.11	33.16 ± 1.03###	41.95 ± 2.98*	33.17 ± 3.53	39.30 ± 4.54	41.92 ± 1.69*
PLT (×10^3^/μL)	565.50 ± 17.37	1070.80 ± 0.03###	801.75 ± 45.88***	961.25 ± 79.79	704.00 ± 92.50***	618.50 ± 30.22***

*Note:* The normal (NOR) and negative control (DMBA) groups received 5% ethanol (vehicle); Tamox + DMBA = positive control rats received tamoxifen as standard at 3.3 mg/kg BW; AE + DMBA = rats treated with the aqueous extract of the bark of *Erythrina excelsa* at doses of 75, 150 and 300 mg/kg body weight, respectively. The values represent the means ± SEM (*n* = 8). All rats, except normal animals, were administered DMBA at a dose of 50 mg/kg. Significance in relation to the NOR group: #*p* < 0.05, ###*p* < 0.001. Significance in relation to the DMBA group: *p < 0.05, **p < 0.01, ***p < 0.001.

Abbreviations: GRAN, granulocytes; HCT, hematocrit; HGB, hemoglobin; LYMP, lymphocytes; MONO, monocytes; PLT, platelets; RBC, red blood cells; WBC, white blood cells.

#### 3.3.9 Relative Weight of Organs in Female Wistar Rats After 140 Days

The administration of DMBA induced a significant increase of the relative weight of the liver (*p* < 0.01), kidney (*p* < 0.001), adrenal gland (*p* < 0.001), spleen (*p* < 0.001), and brain (*p* < 0.001), whereas decreasing uterine weight (*p* < 0.05). Tamoxifen significantly reduced uterine (*p* < 0.001), ovarian (*p* < 0.01), and spleen (*p* < 0.05) weights compared with DMBA rats. Treatment with AE significantly (at least *p* < 0.05) decreased spleen weight and increased femur weight across all doses (Table 4).

**TABLE 4:** Effects of *E. excelsa* aqueous extract on relative weight of organs.

Organs	NOR	DMBA	TAMOX	AE75	AE150	AE 300
Heart	2815.18 ± 121.72	2813.48 ± 169.68	3490.74 351	3321.78 ± 250.98	4037.85 ± 439.45**	3374.05 ± 65.86
Uterus	2528.99 ± 190.88	2144.99 ± 181.29#	773.42 ± 61.72***	2259.82 ± 419.62	2531.26 ± 149.13	1944.00 ± 332.38
Liver	28128.35 ± 1549.23	35604.61 ± 1483.35##	29829.15 ± 1960.25	33687.78 ± 3599.47	37519.16 ± 2420.28**	22301.05 ± 2084.54**
Kidneys	4218.16 ± 167.82	6102.85 ± 224.28###	6612.31 ± 415.21	6582.74 ± 139.77	7452.86 ± 830.94	5763.80 ± 83.60
Adrenal glands	202.02 ± 12.82	310.50 ± 20.00###	266.22 ± 18.27	327.15 ± 38.22	342.12 ± 41.92	214.44 ± 16.33
Lungs	8172.37 ± 421.57	7314.96 ± 454.47	7574.39 ± 718.08	9732.82 ± 485.76	8557.00 ± 460.73**	6534.27 ± 265.63
Femur	2777.16 ± 140.54	2476.36 ± 131.61	3351.67 ± 227.98*	3290.98 ± 153.78*	3460.88 ± 460.73**	3307.94 ± 229.09*
Spleen	2924.50 ± 133.08	6447.91 ± 378.05###	4604.63 ± 266.12*	3290.98 ± 153.78*	3721.50 ± 100.39*	3371.92 ± 65.23**
Brain	5242.94 ± 312.58	8294.06 ± 285.93###	7475.52 ± 284.14	6580.55 ± 325.65	7744.01 ± 756.40	5421.34 ± 139.27**
Ovaries	537.11 ± 40.75	554.61 ± 18.53	176.22 ± 22.47**	560.63 ± 87.78	503.74 ± 40.68	470.22 ± 26.92

*Note:* The normal (NOR) and negative control (DMBA) groups received 5% ethanol (vehicle); Tamox + DMBA = positive control rats received tamoxifen as standard at 3.3 mg/kg bw; AE + DMBA = rats treated with the aqueous extract of the bark of *Erythrina excelsa* at doses of 75, 150 and 300 mg/kg body weight, respectively. The values represent the means ± SEM (*n* = 8). All rats, except normal animals, were administered DMBA at a dose of 50 mg/kg. Significance in relation to the NOR group. Significance in relation to the NOR group: #*p* < 0.05, ##*p* < 0.01, ###*p* < 0.001. Significance in relation to the DMBA group: *p < 0.05, **p < 0.01.

## 4 Discussion

Breast cancer causes many deaths worldwide including sub‐Saharan Africa and has become a major problem for both the population and the scientific community [29]. Despite the availability of many drugs targeting this condition, their negative side effects have led to drawbacks. However, there is currently a need for research on natural anti‐tumor substances and one of the main strategies is to study local natural substances according to their traditional uses [30]. This study was conducted to better understand the anticancer potential of *Erythrina excelsa* bark extract on breast cancer. In this context, the present study investigated the chemopreventive and cytotoxic potential of *Erythrina excelsa* aqueous extract (AE) with emphasis on its phytochemical composition, antioxidant activity, and mechanisms of action both in vitro and in vivo.

Phytochemical screening revealed that AE and EE contain a rich profile of secondary metabolites, notably polyphenols, flavonoids, flavonols, and tannins, with polyphenols being the most abundant. These metabolites are widely recognized for their antioxidant and anticancer properties, primarily through neutralization of reactive oxygen species (ROS) [31]. Consistent with this composition, AE exhibited strong antioxidant activity in DPPH and FRAP assays, confirming its capacity to scavenge free radicals and reduce ferric ions, suggesting a potential role in mitigating oxidative stress during carcinogenesis.

In vitro cytotoxicity assays further confirmed the anticancer property of *Erythrina excelsa* stem bark. The aqueous (AE) and ethanolic (EE) extracts exhibited selective cytotoxicity, with lower IC_50_ values in MCF‐7 compared to MDA‐MB‐231 cells, consistent with the greater sensitivity of hormone receptor‐positive cells to phytochemicals targeting estrogen‐related pathways [32]. However, the ethanolic extract (EE) demonstrated heightened levels of cellular toxicity across both cell lines, indicating that ethanol selectively extracts bioactive compounds, including flavonoids and alkaloids, which are present in high concentrations within Erythrina species [33]. These compounds are known to disrupt cancer cell viability via modulation of signaling cascades including PI3K/AKT, NF‐κB, and MAPK [31, 34]. Morphological analysis confirmed that AE induces apoptosis, as evidenced by nuclear condensation, membrane blebbing, and apoptotic body formation. Apoptosis is a desirable mechanism of cancer cell death since it minimizes inflammation compared to necrosis. Several *Erythrina*‐derived compounds such as n‐hexacosanyl isoferulate, tetradecyl isoferulate, and 1‐heneicosanol have been shown to activate mitochondrial apoptotic pathways through caspase‐3 activation [34]. Flow cytometry analysis revealed that AE induces G2/M arrest in MCF‐7 cells, suggesting interference with cell cycle progression. G2/M arrest is commonly triggered by DNA damage or disruption of microtubule dynamics, preventing mitotic entry and favoring apoptotic signaling [35]. Similar effects have been reported with Erythraline, an alkaloid isolated from *Erythrina velutina* which induced caspase‐independent apoptosis and G2/M cell cycle arrest in SiHa cells [36]. However, AE failed to induce significant cell cycle arrest in MDA‐MB‐231 cells, possibly reflecting their more aggressive phenotype and resistance to checkpoint regulation [37]. The 140‐day administration of AE had no significant effect on body weight in treated rats, indicating good tolerability of the extract throughout the experimental period. In contrast, tamoxifen treatment was associated with a reduction in body weight, consistent with its well‐documented anorectic effect. Tamoxifen is known to have an anorectic effect, thereby inhibiting fatty acid synthase expression and activity, leading to hypothalamic accumulation of malonyl‐CoA and disruption of leptin receptor signaling [38]. Importantly, both AE and tamoxifen significantly improved survival in DMBA‐exposed rats, compared with the DMBA‐only group, where 100% tumor incidence and two deaths were recorded. Treatment with AE at different doses, as well as with tamoxifen, markedly reduced tumor incidence, tumor growth, tumor volume, and overall tumor burden. The chemopreventive efficacy of AE is likely linked to its polyphenol‐rich phytochemical composition, as polyphenols are known to exert cytotoxic, anti‐estrogenic effect by binding to estrogen receptors, thereby blocking estrogen‐mediated tumor cell proliferation antiproliferative effects on breast cancer cells [39].

Cancer antigen 15–3 (CA15‐3), a circulating biomarker derived from the MUC‐1 gene, is a glycoprotein commonly expressed in both benign and malignant mammary ductal epithelial cells. Overexpression of CA15‐3 is observed in approximately 90% of breast cancer cases and is widely used to monitor disease progression and therapeutic response. In the present study, DMBA exposure significantly elevated serum CA15‐3 levels, consistent with enhanced tumor burden and aggressive cellular proliferation. Treatment with tamoxifen, as expected, resulted in a marked reduction of CA15‐3 levels, confirming its efficacy in suppressing tumor progression. *Erythrina excelsa* aqueous extract (AE) similarly reduced CA15‐3 levels at all tested doses, with the most pronounced effect observed at 300 mg/kg body weight. This decrease paralleled the reduction in tumor mass and burden, strongly suggesting that AE attenuates tumorigenesis through mechanisms that ultimately downregulate CA15‐3 expression. Given the polyphenol‐rich composition of AE, its activity is likely mediated by free radical scavenging and redox modulation, resulting in cytotoxicity against tumor cells and suppression of cancer‐associated protein expression [40]. The comparable efficacy of AE to tamoxifen in reducing both tumor growth and CA15‐3 levels underscores its potential as a natural chemopreventive agent.

Under normal physiological conditions, tissues produce free radicals that are usually neutralized by antioxidant agents. However, exposure to chemicals such as DMBA can disrupt these protective mechanisms, causing an imbalance between the levels of pro‐oxidants and antioxidants, leading to reactive oxygen species (ROS) accumulation [41]. Elevated ROS levels can damage cellular macromolecules, particularly polyunsaturated fatty acids in membranes, leading to lipid peroxidation and subsequent DNA damage, events that play a pivotal role in carcinogenesis [42]. The AE treatment resulted in a significant reduction in MDA levels, while concomitantly restoring the activities of key antioxidant enzymes, including superoxide dismutase (SOD), catalase, and glutathione (GSH). These enzymes play a pivotal role in the detoxification of superoxide anions and hydrogen peroxide, thereby ensuring the maintenance of redox homeostasis and protecting against ROS‐mediated cellular injury [43]. The enhancement of antioxidant defences in response to AE treatment suggests that the extract preserves membrane integrity by preventing lipid peroxidation and attenuating the release of lytic enzymes and pro‐inflammatory mediators [44].

This study investigated the levels of cytokines, including IL‐6, TNF‐α, IL‐12, and IFN‐γ, which are critically involved in immune regulation and inflammatory signalling pathways such as N‐terminal kinase c‐Jun (JNK), caspases, and nuclear factor kappa B (NF‐κB) [45]. Elevated IFN‐γ levels have been correlated with tumor growth, suggesting that its suppression may be beneficial in cancer management [45]. Tamoxifen and AE significantly decreased the TNF‐α, IL‐6, IL‐12 and IFN‐γ activities compared to the DMBA group. The downregulation of TNF‐α and IL‐6 is particularly relevant, as both cytokines enhance NF‐κB and JNK transcriptional activity, facilitating epithelial‐to‐mesenchymal transition (EMT) and contributing to tumorigenesis and hormone therapy resistance [46]. By attenuating these pro‐inflammatory mediators, AE likely interferes with EMT induction, thereby limiting tumor progression. Furthermore, suppression of IFN‐γ may prevent aberrant JAK2/STAT1 activation, reducing the risk of DNA damage and oncogenic signalling.

From a toxicological perspective, alterations in relative organ weights are widely recognized as sensitive indicators of potential systemic side effects induced by toxic substances [47]. Following DMBA administration, significant increases were observed in the wet weights of the liver, lungs, ovaries, and kidneys, whereas the adrenal glands and spleen remained unaffected. The pronounced increase in liver wet weight following DMBA administration correlated with elevated serum ALT and AST activities, biomarkers indicative of hepatocellular injury and impaired liver function, while DMBA exposure also led to a significant rise in plasma creatinine levels, reflecting renal dysfunction [48]. Creatinine is normally cleared efficiently by renal glomerular filtration; thus, its accumulation indicates a decline in renal clearance capacity. Treatment with AE attenuated the DMBA‐induced increases in liver and kidney weights, while also normalizing ALT, AST, and creatinine levels, suggesting a protective effect of the extract on both hepatic and renal function. The hematopoietic system is highly sensitive to xenobiotic exposure and serves as a reliable indicator of overall physiological status. In this study, hematological evaluation provided further evidence of systemic toxicity. DMBA‐exposed animals exhibited a significant rise in mean corpuscular volume (MCV), along with reductions in red blood cell counts, hemoglobin concentration, and hematocrit levels. Such alterations are indicative of anemia, a frequent complication in both cancer progression and anticancer therapy, which adversely affects energy levels, quality of life, and clinical outcomes in breast cancer patients [49]. Importantly, AE treatment mitigated these hematological disruptions by restoring red blood cell and platelet counts while reducing MCV levels, suggesting its capacity to modulate hematopoietic homeostasis and immune balance [50].

To address the shortcomings of this study, it would have been interesting to assess the effects of AE on caspase 9 and on anti‐apoptotic proteins such as Bcl‐XL and Bcl‐2, in order to confirm that it acts via intrinsic apoptosis. Furthermore, although in vitro studies have shown that AE is more active on MCF‐7 cells than on MDA‐MB‐231 cells, and although the cancer model used in vivo replicated an estrogen‐dependent cancer type, an assessment of the extract's anti‐estrogenic mechanisms would have provided further evidence. A standardised chromatographic analysis of the AE extract, combined with *in silico* studies, could also be carried out to formulate hypotheses regarding the ADME profile as well as the molecular mechanisms of AE.

## 5 Conclusion

This study examined the potential anticancer effects of the extracts *Erythrina excelsa* stem bark both in vitro and in vivo. Phytochemical profiling revealed that the extracts are particularly rich in polyphenols, flavonoids, and tannins, which are closely associated with strong free radical scavenging and reducing activities. In vitro, the extracts effectively inhibited the growth and proliferation of breast cancer cells (MCF‐7 and MDA‐MB‐231) through dose‐dependent cytotoxicity and apoptosis induction, as well as G2/M phase arrest. In vivo, administration of the *Erythrina excelsa* aqueous extract significantly reduced tumor incidence, volume, and serum CA 15–3 levels in DMBA‐induced mammary carcinogenesis. Simultaneously, it restored redox homeostasis, attenuated inflammatory cytokine production (IFN‐γ, TNF‐α, IL‐6, and IL‐12), and preserved hematological and biochemical parameters. Overall, the extract exerted these chemopreventive effects without evident systemic toxicity, highlighting *E. excelsa* as a valuable source of bioactive compounds with potent antioxidant and anti‐inflammatory properties and supporting its potential as a natural chemopreventive agent against breast cancer.

## Author Contributions

**Derek Tantoh Ndinteh:** conceptualization, writing – review and editing, supervision, validation. **Merline Nguedia Ymele:** methodology, writing – review and editing, data curation. **Jonas Walantini:** conceptualization, investigation, writing – original draft, methodology. **Dieudonné Njamen:** writing – review and editing, validation, supervision, resources. **Roland Nhouma Rebe:** methodology, writing – review and editing, data curation. **Stéphane Zingue:** conceptualization, writing – original draft, validation, data curation, supervision, resources. **Joël Abel Gbaweng Yaya:** writing – review and editing, data curation, resources. **Marius Trésor Kemegne Sipping:** writing – review and editing, data curation, methodology.

## Funding

The authors have nothing to report.

## Consent

The authors have nothing to report.

## Conflicts of Interest

The authors declare no conflicts of interest.

## Data Availability

Any supplementary data for the reproducibility of this study are available upon request from the corresponding author (stephane.zingue@fmsb-uy1.cm).
